# Differential effects of glucagon-like peptide-1 receptor agonists on heart rate

**DOI:** 10.1186/s12933-016-0490-6

**Published:** 2017-01-13

**Authors:** Martin Lorenz, Francesca Lawson, David Owens, Denis Raccah, Christine Roy-Duval, Anne Lehmann, Riccardo Perfetti, Lawrence Blonde

**Affiliations:** 1 R&D Diabetes Division, Sanofi-Aventis Deutschland GmbH, Industrial Park Höchst, Bldg. H831, 65926 Frankfurt am Main, Germany; 2 R&D Diabetes Division, Sanofi, Bridgewater, NJ USA; 3 Institute of Life Sciences College of Medicine, Swansea University, Swansea, UK; 4 University Hospital Sainte-Marguerite, Marseille, France; 5 R&D Diabetes Division, Sanofi, Chilly-Mazarin, France; 6 R&D Clinical Sciences & Operations, Sanofi-Aventis Deutschland GmbH, Frankfurt am Main, Germany; 7 Global Medical Affairs, Sanofi, Bridgewater, NJ USA; 8 Department of Endocrinology, Frank Riddick Diabetes Institute, Ochsner Medical Center, New Orleans, LA USA

**Keywords:** Type 2 diabetes mellitus, Glucagon-like peptide-1 receptor agonist, Heart rate

## Abstract

While glucagon-like peptide-1 receptor agonists (GLP-1 RAs) are known to increase heart rate (HR), it is insufficiently recognized that the extent varies greatly between the various agonists and is affected by the assessment methods employed. Here we review published data from 24-h time-averaged HR monitoring in healthy individuals and subjects with type 2 diabetes mellitus (T2DM) treated with either short-acting GLP-1 RAs, lixisenatide or exenatide, or long-acting GLP-1 RAs, exenatide LAR, liraglutide, albiglutide, or dulaglutide (N = 1112; active-treatment arms). HR effects observed in two independent head-to-head trials of lixisenatide and liraglutide (N = 202; active-treatment arms) are also reviewed. Short-acting GLP-1 RAs, exenatide and lixisenatide, are associated with a transient (1–12 h) mean placebo- and baseline-adjusted 24-h HR increase of 1–3 beats per minute (bpm). Conversely, long-acting GLP-1 RAs are associated with more pronounced increases in mean 24-h HR; the highest seen with liraglutide and albiglutide at 6–10 bpm compared with dulaglutide and exenatide LAR at 3–4 bpm. For both liraglutide and dulaglutide, HR increases were recorded during both the day and at night. In two head-to-head comparisons, a small, transient mean increase in HR from baseline was observed with lixisenatide; liraglutide induced a substantially greater increase that remained significantly elevated over 24 h. The underlying mechanism for increased HR remains to be elucidated; however, it could be related to a direct effect at the sinus node and/or stimulation of the sympathetic nervous system, with this effect related to the duration of action of the respective GLP-1 RAs. In conclusion, this review indicates that the effects on HR differ within the class of GLP-1 RAs: short-acting GLP-1 RAs are associated with a modest and transient HR increase before returning to baseline levels, while some long-acting GLP-1 RAs are associated with a more pronounced and sustained increase during the day and night. Findings from recently completed trials indicate that a GLP-1 RA-induced increase in HR, regardless of magnitude, does not present an increased cardiovascular risk for subjects with T2DM, although a pronounced increase in HR may be associated with adverse clinical outcomes in those with advanced heart failure.

## Background

The injectable glucagon-like peptide-1 receptor agonists (GLP-1 RAs) are widely used in the management of type 2 diabetes mellitus (T2DM) [1]. These agents preferentially lower fasting and postprandial hyperglycemia according to their pharmacokinetic (PK) characteristics, while also being associated with body weight loss [1–6]. GLP-1 RAs exhibit a broad range of effects on the cardiovascular system that are independent of changes in blood glucose. The majority of these are cardio-protective, including lowering systolic and diastolic blood pressure [5, 6] and attenuating hypotension induced by intraduodenal glucose infusion [7], lowering plasma cholesterol level [5, 8] and ischemia–reperfusion injury [9], and delaying the progression of atherosclerotic disease [9]. GLP-1 RAs have also been suggested to be nephroprotective [10], even though conflicting data on renal sodium and water handling have been reported [10–12].

GLP-1 RAs induce an increase in heart rate (HR), which, theoretically, represents a safety concern, as this is considered to be associated with higher cardiovascular risk [13, 14]. Initially it was believed that this positive chronotropic effect was not clinically relevant and was of a similar magnitude for all GLP-1 RAs. A meta-analysis of Phase 3 trials reported HR increases in the range of 1–4 beats per minute (bpm) for exenatide twice daily (BID), exenatide long-acting release (LAR), and liraglutide [15]. In these studies, HR was assessed by pulse rate measurements at a single time point. In order to allow a more sensitive differentiation of GLP-1 RA-dependent HR effects, the current manuscript presents HR data derived from ECG measurements and ambulatory blood pressure monitoring at multiple time points over 24 h.

Six injectable GLP-1 RAs are currently approved for the treatment of T2DM in Europe and in the USA. Exenatide is a synthetic version of exendin-4, a potent GLP-1 RA isolated from the venom of the Gila monster, and approved for BID administration, with exenatide LAR being a microsphere formulation of exendin-4 that enables once weekly (QW) administration [3]. Lixisenatide is structurally related to exendin-4 (six lysine residues have been added to the C terminus, and one proline in the C-terminal region has been deleted) but can be administered once daily (QD). Liraglutide is in close structural homology to native GLP-1 and is conjugated to a palmitic acid to prolong its half-life for QD administration [3]. Albiglutide comprises two copies of GLP-1 fused as tandem repeats to the N terminus of albumin [3], while dulaglutide is a recombinant fusion protein consisting of two GLP-1 peptides covalently linked to a human IgG4-Fc heavy-chain variant [3]. These formulations prolong the PK profile of both of these preparations to allow QW administration [3]. According to their respective PK profiles, exenatide BID and lixisenatide can be categorized as short-acting agents (each has a half-life of 3–5 h) compared with the long-acting agents liraglutide, exenatide LAR, albiglutide, and dulaglutide, which have half-lives ranging from 12 h to several days [16].

Based on the different molecular structures and PK characteristics of the GLP-1 RAs and the presence of GLP-1 receptors on the sinoatrial node, differential effects on HR may be expected. Therefore, the aim of this review of published data is to compare the magnitude and duration of the effects on HR induced by various GLP-1 RAs, both in healthy individuals and in those with T2DM, utilizing individual 24-h HR measurements from clinical trials, including head-to-head comparison data.

## Data search

PubMed searches were performed using relevant terms, including ‘GLP-1’, ‘GLP-1 receptor agonists’, ‘Exenatide BID’, ‘Lixisenatide’, ‘Liraglutide’, ‘Albiglutide’, ‘Dulaglutide’, ‘Exenatide LAR’, ‘heart rate’, and ‘pulse rate’. Priority for inclusion in this manuscript was awarded to studies that measured HR over a full 24-h period by serial ECG monitoring or those using ambulatory blood pressure measurements including serial or continuous HR detection. Two trials fulfilling these criteria directly compared the short-acting GLP-1 RA lixisenatide with the long-acting GLP-1 RA liraglutide [17, 18]. Moreover, relevant data from product monographs were also reviewed.

## Effects of short-acting GLP-1 RAs on HR

### Exenatide BID

**Fig. 1:**
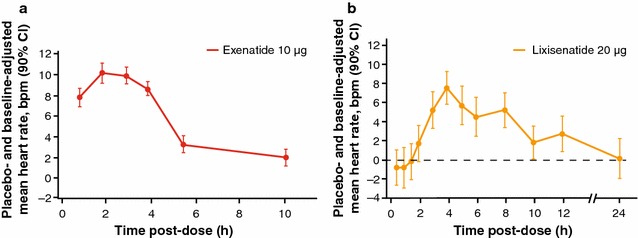
Changes from baseline in the placebo-adjusted mean HR as a function of time for **a** exenatide 10 µg [20] and **b** lixisenatide 20 µg QD (Sanofi, data on file). *bpm* beats per minute, *CI* confidence interval, *HR* heart rate, *QD* once daily. **a** Reproduced with permission from AstraZeneca

In a QT study using serial ECG monitoring, exenatide 10 µg QD was found to result in a transient HR increase of 10 bpm in 62 healthy individuals 2 h after a single injection. The effect faded over time, with HR returning to baseline levels after approximately 10 h (Fig. 1a) [19, 20]. The peak HR increase mirrored the PK profile, which shows that the maximum plasma concentration of exenatide is reached 2 h after injection [20]. In a second study in 28 patients with T2DM, the mean 24-h HR was assessed after 12 weeks’ treatment with exenatide 10 µg BID (5 µg BID initiation dose for 4 weeks followed by 10 µg BID). HR averaged over 24 h increased by 2 bpm with exenatide BID compared with a decrease of 1 bpm for placebo (n = 26) at the end of the study period [21].

### Lixisenatide

In a QT study, HR data from serial ECG measurements in healthy individuals receiving lixisenatide 20 µg QD for 28 days (N = 68; patients with evaluable data n = 61; dose titration: 10 and 15 µg during weeks 1 and 2, respectively; 20 µg during weeks 3 and 4) demonstrated a maximum HR increase of 7.3 bpm 4 h post-dosing [22]. As shown in Fig. 1b, the changes in HR were transient and reverted to baseline after 12 h (Sanofi, data on file; data available on request). The mean 24-h HR increase adjusted for baseline and placebo values was 1.3 bpm [22].

## Effect of long-acting liraglutide QD on HR

**Fig. 2:**
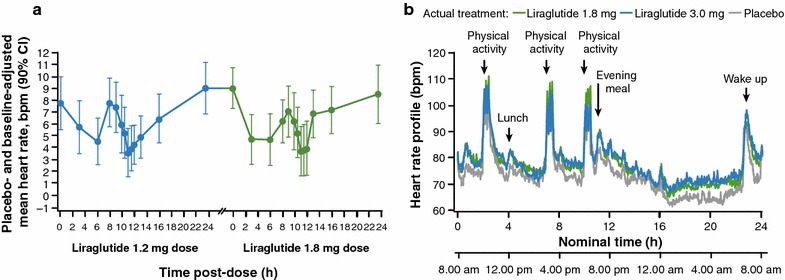
**a** Changes from baseline in the placebo-adjusted mean HR as a function of time for liraglutide 1.2 and 1.8 mg QD [23]. **b** 24-h HR profiles for liraglutide 1.8 and 3.0 mg, and placebo QD [24]. *bpm* beats per minute, *CI* confidence interval*, HR* heart rate, *QD* once daily. Reproduced with permission from Novo Nordisk and the FDA

A 24-h time-averaged increase in mean placebo- and baseline-adjusted HR of 7–8 bpm was reported following liraglutide 1.2 and 1.8 mg QD (titrated in 0.6-mg weekly steps) using serial ECG monitoring at the end of the second and third weeks (after the seventh and final dose) of liraglutide (N = 51). Notably, the elevation in HR persisted through the 24-h measurement period for both doses, and was characterized by an initial increase followed by a decline and then a second more persistent increase (Fig. 2a) [23].

In a second study [24, 25], the effect of liraglutide on HR was investigated by 24-h continuous HR monitoring in obese individuals (3 mg n = 32; 1.8 mg n = 30) without diabetes following 5 weeks’ treatment (titrated from 0.6 mg subcutaneously and increased in increments of 0.6 mg per week, up to end doses of 1.8 and 3.0 mg). HR increased during the day and at nighttime with both doses compared with placebo (Fig. 2b), with the overall 24-h HR increased by 6–7 bpm. The 3.0-mg dose (approved for treatment in obese subjects) was associated with a further 1-bpm HR increase over 24 h compared with the 1.8-mg dose. The HR increases with both liraglutide doses were more pronounced during nighttime (7.0–8.9 bpm) compared with those seen during the day (4.3–4.6 bpm).

Similar results were obtained in a third placebo-controlled study of liraglutide 1.8 mg, which also employed 24-h ambulatory HR monitoring in overweight or obese hypertensive subjects with T2DM (n = 17). At 3 weeks’ follow-up, HR with liraglutide was significantly elevated compared with placebo (n = 17) for the whole 24-h period, day- and nighttime (least squares mean differences were 5.2, 4.6, and 7.3 bpm, respectively) [26].

## Comparison of short- vs. long-acting GLP-1 RAs on HR: lixisenatide vs. liraglutide

**Fig. 3:**
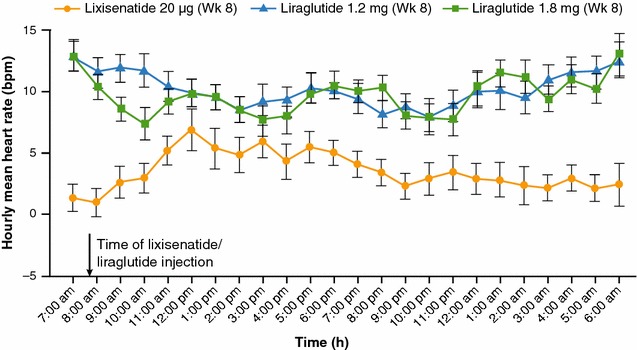
Changes from baseline in 24-h mean HR, as assessed by ambulatory blood pressure monitoring, for lixisenatide vs. liraglutide. *bpm* beats per minute, *HR* heart rate. Reproduced from Meier et al. [17]. [American Diabetes Association, 2015. Copyright and all rights reserved. Material from this publication has been used with the permission of American Diabetes Association.]

The effects on HR for the short-acting RA lixisenatide vs. the long-acting GLP-1 agent liraglutide were analyzed in two independent head-to-head comparison studies [17, 18]. In the first trial, lixisenatide 20 µg QD (n = 46) and liraglutide 1.2 (n = 44) and 1.8 mg QD (n = 46) were compared following 8 weeks’ treatment in 142 subjects with T2DM not adequately controlled with insulin glargine with or without metformin [17]. Both GLP-1 RAs were administered in the morning and the approved titration schemes were applied. 24-h HR was assessed using standard ambulatory blood pressure monitoring at baseline and after 8 weeks (Fig. 3).

**Fig. 4:**
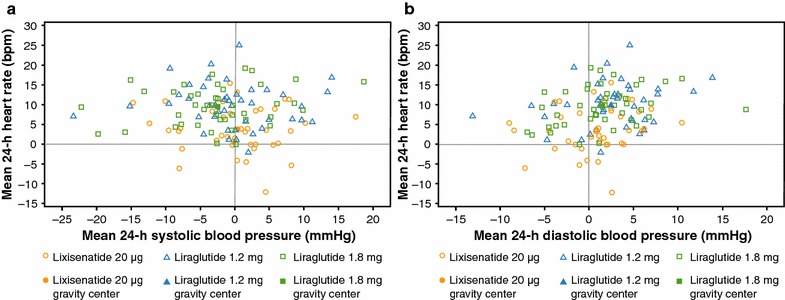
Relationship between the effect of lixisenatide on HR and **a** systolic blood pressure and **b** diastolic blood pressure. Baseline is defined as the 24-h profile on week –1 (day –2/–1) determined as overall mean. *bpm*, beats per minute, *HR* heart rate

Mean (standard error [SE]) increases in 24-h HR from baseline were 3.3 (1.3) bpm for lixisenatide 20 µg vs. 9.3 (1.2) and 9.2 (1.3) bpm for liraglutide 1.2 and 1.8 mg, respectively (mean differences vs. lixisenatide were 6.0 and 5.8 bpm for liraglutide 1.2 and 1.8 mg, respectively; p < 0.0001) [17]. Further analysis (Sanofi, data on file; data available on request) revealed a 4-bpm increase for lixisenatide vs. an approximate 9-bpm increase for both liraglutide 1.2 and 1.8 mg during the daytime. At nighttime, lixisenatide increased HR by 2.2 bpm compared with approximately 10 bpm for both liraglutide doses (Table 1). None of the HR increases correlated with a decrease in either systolic or diastolic blood pressure (Fig. 4).

**Table 1:** Daytime and nighttime HR changes (bpm)

Daytime	Lixisenatide 20 μg (n = 40)	Liraglutide 1.2 mg (n = 40)	Liraglutide 1.8 mg (n = 41)
At baseline	71.0	69.3	70.0
At week 8	74.7	79.3	79.4
LS mean (SE) change from baseline	3.67 (1.42)	9.41 (1.31)	9.10 (1.38)
LS mean (SE) difference vs. lixisenatide		5.74 (1.23)	5.43 (1.20)
Difference vs. lixisenatide: p value		<0.0001	<0.0001

Nighttime	Lixisenatide 20 μg (n = 42)	Liraglutide 1.2 mg (n = 43)	Liraglutide 1.8 mg (n = 44)
At baseline	67.1	65.1	66.2
At Week 8	69.7	75.7	76.7
LS mean (SE) change from baseline	2.20 (1.47)	9.97 (1.37)	10.12 (1.45)
LS mean (SE) difference vs. lixisenatide		7.78 (1.26)	7.92 (1.24)
Difference vs. lixisenatide: p value		<0.0001	<0.0001

Daytime and nighttime HR changes (bpm) following 8 weeks’ treatment with lixisenatide vs. liraglutide in subjects with T2DM are shown. Please note that n numbers are for the number of patients for whom HR data were available

*bpm* beats per minute, *HR* heart rate, *LS* least squares, *SE* standard error, *T2DM* type 2 diabetes mellitus

**Fig. 5:**
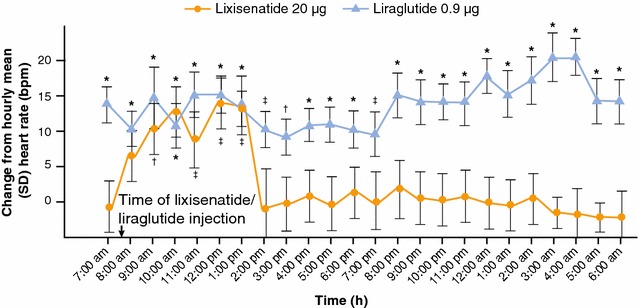
Diurnal profile of HR changes in subjects with T2DM at baseline and after treatment with liraglutide or lixisenatide [18]. Data are mean (SD). ^‡^p ≤ 0.05; ^†^p ≤ 0.01; *p ≤ 0.001 vs. baseline. *bpm* beats per minute, *HR* heart rate, *SD* standard deviation, *T2DM* type 2 diabetes mellitus. Reproduced from Nakatani et al. [18]. [American Diabetes Association, 2016. Copyright and all rights reserved. Material from this publication has been used with the permission of American Diabetes Association.]

A second head-to-head comparison trial between lixisenatide and liraglutide was performed in 60 subjects with T2DM [18]. In this study, a 24-h Holter ECG recording was performed at baseline, after the maximum daily dose for each drug (lixisenatide 20 µg, liraglutide 0.9 mg) had been administered for at least 1 week following completion of the up-titration period. In the lixisenatide-treated group, a significant increase in HR was observed only at 5 h post-dosing, and the mean HR per day remained unchanged (69.1 ± 8.6 to 71.7 ± 10.6 bpm; p = 0.172; Fig. 5). In contrast, in the liraglutide-treated group, the mean daily HR increased significantly from baseline at all times (66.5 ± 10.2 to 79.7 ± 10.5 bpm; p = 0.00021; Fig. 5).

## Effects on HR of the long-acting QW GLP-1 RAs: exenatide LAR, albiglutide and dulaglutide

### Exenatide LAR

A thorough QT study evaluated the effects of steady-state therapeutic (253 and 399 pg/ml) and supra-therapeutic (627 pg/ml) plasma concentrations of exenatide following continuous intravenous infusion of exenatide LAR to healthy individuals (N = 86; patients with evaluable data, n = 74) over three 24-h periods, each separated by a washout period of at least 5 days [27]. HR was monitored continuously throughout each infusion period. At these two concentrations of exenatide, the mean HR increased by up to 16.8 bpm compared with an increase of 5.5 bpm with placebo. In a second QT study, the effect of a QW injection of exenatide LAR on HR in 148 subjects with T2DM was evaluated after 14 and 30 weeks’ treatment [28]. In this study, a mean increase in 24-h HR of 3.6 and 3.5 bpm was observed at weeks 14 and 30, respectively.

### Albiglutide

The effect of albiglutide QW on HR was investigated in a study involving ECG measurements in 85 healthy individuals after 6 weeks’ treatment (albiglutide was administered at a dose of 30 mg [initiation dose] for 2 weeks followed by 50 mg [optional dose based on individual glycemic response] for 4 weeks) [29]. The placebo-corrected increase in mean HR from baseline at the end of the study (day 39) was up to 3 and 6–8 bpm for the 30- and 50-mg dose, respectively.

### Dulaglutide

The HR effects of dulaglutide 0.75 mg and 1.5 mg were investigated using ambulatory blood pressure and HR measurements in 505 subjects with T2DM following 26 weeks’ treatment [30]. Dulaglutide 0.75 mg (n = 251) resulted in a mean increase in 24-h HR of 1.3 bpm, whereas a 3.5-bpm increase in HR was observed at the end of the study for the 1.5-mg dose (n = 254). When comparing effects during the daytime and at nighttime after 26 weeks’ treatment, the HR increase compared with placebo was only significant during the nocturnal period for the lower dulaglutide 0.75-mg dose. For dulaglutide 1.5 mg, similar HR increases compared with placebo were reported during both diurnal and nocturnal periods.

## Discussion

This review evaluates the effect on HR of six approved GLP-1 RAs, categorized into a short-acting sub-class with half-lives of 3–5 h (exenatide BID and lixisenatide) and a long-acting sub-class with half-lives ranging from 12 h to several days (liraglutide, exenatide LAR, albiglutide and dulaglutide) [16].

A recent meta-analysis of Phase 3 trials reported increases in HR, based on single time point assessments, in the range of 1–4 bpm with both long- and short-acting GLP-1 RAs [15]. However, the present review, which relies specifically on 24-h-averaged HR data, demonstrates that the degree of this positive chronotropic effect was underestimated when assessed by single time point pulse rate measurements. When evaluated with more sensitive and reliable 24-h continuous monitoring and in thorough QT studies, two of the long-acting GLP-1 RAs, liraglutide and albiglutide, were shown to cause a pronounced increase in the 24-h time-averaged mean HR from baseline of up to 6–10 bpm, while for dulaglutide and exenatide LAR, the increase in 24-h mean HR was 3–4 bpm [23, 24, 27–30]. The two short-acting GLP-1 RAs, exenatide BID and lixisenatide, both caused a maximum HR increase of 8–10 bpm 2–4 h after injection, which returned to baseline levels after approximately 10–12 h (Sanofi, data on file [available on request [22] ] and [19, 20]), with a mean 24-h HR increase of approximately 1–2 bpm for both preparations.

A limitation of the current review is that it focused on indirect comparisons of 24-h continuous monitoring studies that differed in terms of study population (e.g., obese or not-obese, ambulatory or not-ambulatory), study treatment (e.g., continuous intravenous infusion, or oral administration) and timing of assessment after variable duration of study treatment. To overcome this limitation, two independent head-to-head comparisons between short-acting lixisenatide and long-acting liraglutide in subjects with T2DM were also reviewed. These direct comparisons allowed a more sensitive differentiation of HR effects, and confirmed that lixisenatide induces a significantly smaller increase in mean 24-h HR compared with liraglutide (4 vs. 9 bpm; Figs. 3, 5) [17, 18].

It could be hypothesized that there is an association between the PK of the various GLP-1 RAs, the duration of receptor activation/occupancy and the extent of the HR effect. Brief receptor occupancy with short-acting GLP-1 RAs could explain the transient effect on HR, while prolonged receptor occupancy could explain the more pronounced HR effect observed with some long-acting GLP-1 RAs. Furthermore, the longer half-life resulting in receptor activation over 24 h would be in line with the increased HR during the diurnal and nocturnal periods reported for liraglutide and dulaglutide [24, 30]. However, this relationship does not account for the differences in HR increases within the sub-class of the long-acting agents. The differential effects of these molecules could be explained by differences in their affinity for the GLP-1 receptor, especially variations in their binding kinetics, e.g., on/off rates. Furthermore, the differences between the agents in terms of the duration of their effect may be due to tachyphylaxis and/or receptor desensitization.

The underlying mechanism for the increased HR has not yet been fully elucidated. It was initially postulated to be a compensatory mechanism to the blood pressure lowering effect of GLP-1 RAs that was possibly mediated by their vasodilatory effect [9, 31] and/or increased urinary sodium excretion [32]. However, no association between HR and blood pressure was observed in the head-to-head comparison trial of liraglutide and lixisenatide [17] (Fig. 4). It is more likely that increased HR results from a direct GLP-1 receptor-mediated effect on the endogenous sinoatrial pacemaker node of the heart, as GLP-1 receptors are expressed in the human heart in myocytes in the sinoatrial node [33]. Such a direct GLP-1 mechanism is supported by studies using native GLP-1 and exenatide in healthy people and patients with T2DM showing that the acute increase of HR was not preceded by a decrease in blood pressure, was not associated with changes in plasma levels of adrenaline or noradrenaline, and was independent of GLP-1 glucoregulatory effects [11, 34, 35]. A further possibility is that the positive chronotropic effect could be, at least partially, mediated by sympathetic nervous system enhancement, related to the inhibition of the autonomic nervous system [18, 36, 37]. In subjects with T2DM, 24-h Holter ECG and power spectrum analysis of HR variability were conducted before and after liragutide or lixisenatide administration. In the liraglutide group, significant differences were observed in the low (0.04–0.15 Hz)/high (0.2–0.4 Hz) frequency ratio; however, no changes in the low/high frequency ratio were found in the lixisenatide group [18]. This mechanism of action would explain the more marked increase in HR induced by liraglutide in those subjects with T2DM at night when parasympathetic activity is predominant. Diminished parasympathetic modulation of the heart resulting in increased HR was also observed in response to intracerebroventricular infusion of exendin-4 in mice [36].

A substantial volume of data indicates that an elevated HR is independently associated with increased cardiovascular morbidity and mortality in the general population, in subjects with cardiovascular diseases, and those with diabetes [13, 14, 38, 39]. In the action in diabetes and vascular disease (ADVANCE) trial, a study involving 11,140 subjects with T2DM who were followed for a median duration of 4.4 years, a higher HR was associated with a significantly increased risk of all-cause mortality, cardiovascular death, and major cardiovascular events (death, nonfatal acute myocardial infarction [AMI] or nonfatal stroke) [39]. In the same study population, a higher HR was also associated with a greater incidence of new-onset or progressive nephropathy and retinopathy [38]. It is unclear whether a higher HR is directly responsible for the increased cardiovascular risk. However, in support of a causal relationship, lowering of HR by ivabradine, a selective inhibitor of the sinus node I(f) channel, devoid of any activity on blood pressure, cardiac contractility, atrioventricular conduction or ventricular repolarization, decreased the risk of cardiovascular death or hospitalization due to heart failure (HF) in subjects with HF, which is a debilitating condition highly sensitive to changes in HR [40].

HF is one of the most common complications of diabetes, and patients with diabetes are at a higher risk of developing HF [41]. Also, diabetes adversely affects the outcomes of subjects with HF, substantially decreasing their survival rate [42]. Achieving good glycemic control in subjects with T2DM and HF is difficult, as several antihyperglycemic agents have been reported to increase the risk of hospitalization due to HF [43–45]. GLP-1 RA treatment might be expected to improve HF outcome by augmenting glucose uptake by the myocardium, a potentially important mechanism in HF as fatty acid metabolism is down-regulated, and adenosine triphosphate synthesis is more dependent on glucose. However, in a recent placebo-controlled crossover study to evaluate the effects of liraglutide in subjects with T2DM and coronary artery disease undergoing dobutamine stress echocardiography (DSE), liraglutide did not improve the systolic function of the left ventricle during DSE or patients’ exercise capacity [46]. The National Heart, Lung and Blood Institute Heart Failure Clinical Research Network designed the Functional Impact of GLP-1 for Heart Failure Treatment (FIGHT) study (NCT01800968) to test the hypothesis that liraglutide would improve myocardium bioenergetics leading to post-hospitalization clinical stability in subjects with advanced HF and reduced left ventricular ejection fraction, with and without diabetes [47]. The study results were published recently [48] and indicated that liraglutide did not improve clinical stability but was rather associated with a numerical but not statistically significant increase in the composite of death or HF hospitalization compared with placebo. Subjects who received liraglutide were also reported to have a higher incidence of arrhythmia vs. placebo (17 vs. 11%, respectively). Similar findings were observed in the Effect of LIraglutide on left VEntricular function in chronic heart failure patients with and without type 2 diabetes (LIVE) study [49, 50]. The trial randomized 241 patients with chronic HF with reduced left ventricular ejection fraction to receive liraglutide or placebo. After 24 weeks’ treatment, there were no significant differences between the groups for the primary endpoint of change in left ventricular ejection fraction, regardless of whether the patients had comorbid diabetes or not. However, patients treated with liraglutide suffered numerically more serious adverse cardiac events than those on placebo (12 vs. 3, respectively; p = 0.04). It is not known whether these adverse clinical outcomes could have been mediated by the pronounced and prolonged increase in HR induced by liraglutide.

The results of the Liraglutide Effect and Action in Diabetes: Evaluation of Cardiovascular Outcome Results (LEADER) trial were published recently [51]. In this randomized trial of more than 9300 subjects with T2DM and high cardiovascular risk, liraglutide was seen to decrease the incidence of the composite cardiovascular primary endpoint (occurrence of death from cardiovascular causes, nonfatal myocardial infarction, or nonfatal stroke) compared with placebo (13.0 and 14.9% in the liraglutide and placebo group, respectively; hazard ratio [HR]: 0.87; 95% confidence interval [CI] 0.78, 0.97). Liraglutide was also associated with a decrease in death from cardiovascular causes and death from any cause, and did not increase the risk of hospitalization due to HF in the overall patient population. No analysis was conducted in the subgroup of patients with HF at baseline (approximately 14%) to determine whether treatment with liraglutide was associated with any change of severity of HF and/or cardiovascular mortality.

The results of the Evaluation of LIXisenatide in Acute coronary syndrome (ELIXA) trial, the first cardiovascular outcomes trial for the short-acting GLP-1 RA lixisenatide, were also published recently [52]. This placebo-controlled study in more than 6000 subjects with T2DM and acute coronary syndrome demonstrated the cardiovascular safety of chronic exposure to lixisenatide. Following a median follow-up of 2.1 years, the risk of suffering a major cardiovascular adverse event (death, nonfatal AMI, nonfatal stroke or hospitalization for unstable angina) was similar in both the lixisenatide and the placebo arms (HR 1.02; 95% CI 0.89, 1.17). Furthermore, lixisenatide showed a consistent neutral effect on all individual components of the primary and secondary endpoints. Importantly, lixisenatide did not increase the risk of hospitalization for HF compared with placebo. The percentages of subjects who were hospitalized for HF were 4.0 and 4.2% in the lixisenatide and placebo group, respectively (HR 0.96; 95% CI 0.75, 1.23).

While LEADER and ELIXA were similar in terms of their overall design, there were some major differences between the patient populations enrolled in the two studies. Most notably, the patients enrolled in LEADER had chronic cardiovascular risk (either a pre-existing cardiovascular condition or a high risk for cardiovascular disease), while only those individuals who were within 180 days post-acute coronary event and, hence, at the highest risk of a further cardiovascular event, were included in ELIXA. As a result of the different event rates in the two studies, the median durations of follow-up were also substantially different (3.8 years in LEADER vs. 2.1 years in ELIXA). Reduction of atherosclerosis requires long-term treatment [53], hence longer trials are better suited to show a reduction in risk of myocardial infarction or other atherosclerotic complications of diabetes. Furthermore, several key baseline characteristics were also different between the two study populations. For example, mean ± standard deviation glycated hemoglobin levels were 8.7 ± 1.5% in LEADER and 7.7 ± 1.3% in ELIXA. As these and other differences between ELIXA and LEADER may have affected the overall results of the two studies, comparisons between them and extrapolations from one study to another should be made with caution.

Overall, the findings from these two recently completed cardiovascular outcomes trials suggest that the increases in HR associated with the short-acting GLP-1 RA, lixisenatide, or the long-acting GLP-1 RA, liraglutide, do not lead to an increased risk of major adverse cardiac events in patients with T2DM and high cardiovascular risk. Cardiovascular outcomes trials are still ongoing for the other GLP-1 RAs (Table 2) and will provide further insight on the CV effects of these agents.

**Table 2:** Ongoing CVOT for GLP-1 RAs

Drug	CVOT (ClinicalTrial.gov ID)	n	Estimated completion date
Exenatide QW	EXSCEL (NCT01144338)	14,000	2018
Dulaglutide	REWIND (NCT01394952)	9622	2019
Albiglutide	HARMONY outcomes (NCT02465515)	9400	2019

Data in table derived from *ClinicalTrials.gov*

*CVOT* cardiovascular outcome trial, *GLP*-*1 RA* glucagon-like peptide-1 receptor agonist, *QW* once weekly

## Conclusions

In conclusion, this review shows that long-acting GLP-1 RAs induce a pronounced and prolonged increase in 24-h mean HR, while short-acting agents lead to a more transient increase that reverts to baseline levels within a few hours after injection. The underlying mechanisms for increased HR remain to be fully elucidated, but may be related to a direct GLP-1 receptor effect on myocytes in the sinoatrial node of the human heart and/or stimulation of the sympathetic nervous system. According to the recently completed ELIXA and LEADER trials, an increase in HR, regardless of its magnitude, does not appear to increase the cardiovascular risk of individuals with T2DM and with (or at high risk of) cardiovascular disease. However, a pronounced increase in HR may be associated with adverse clinical outcomes in subjects with both T2DM and advanced HF.

## Abbreviations

ADVANCE: action in diabetes and vascular disease
AMI: acute myocardial infarction
BID: twice daily
bpm: beats per minute
CI: confidence interval
CVOT: cardiovascular outcome trial
ELIXA: Evaluation of LIXisenatide in Acute coronary syndrome
FIGHT: Functional Impact of GLP-1 for Heart Failure Treatment
GLP-1 RA: glucagon-like peptide-1 receptor agonist
HF: heart failure
HR: heart rate
LAR: long-acting release
LEADER: Liraglutide Effect and Action in Diabetes: Evaluation of Cardiovascular Outcome Results
LIVE: Effect of LIraglutide on left VEntricular function in chronic heart failure patients with and without type 2 diabetes
LS: least squares
PK: pharmacokinetic
QD: once daily
QW: once weekly
SE: standard error
T2DM: type 2 diabetes mellitus

## References

[1] Inzucchi SE, Bergenstal RM, Buse JB, Diamant M, Ferrannini E, Nauck M, Peters AL, Tsapas A, Wender R, Matthews DR (2015). Management of hyperglycaemia in type 2 diabetes, 2015: a patient-centred approach. Update to a position statement of the American Diabetes Association and the European Association for the Study of Diabetes. Diabetologia.

[2] Drucker DJ, Nauck MA (2006). The incretin system: glucagon-like peptide-1 receptor agonists and dipeptidyl peptidase-4 inhibitors in type 2 diabetes. Lancet.

[3] Lorenz M, Evers A, Wagner M (2013). Recent progress and future options in the development of GLP-1 receptor agonists for the treatment of diabesity. Bioorg Med Chem Lett.

[4] Nauck MA (2011). Incretin-based therapies for type 2 diabetes mellitus: properties, functions, and clinical implications. Am J Med.

[5] Blonde L, Pencek R, Macconell L (2015). Association among weight change, glycemic control, and markers of cardiovascular risk with exenatide once weekly: a pooled analysis of patients with type 2 diabetes. Cardiovasc Diabetol..

[6] Simo R, Guerci B, Schernthaner G, Gallwitz B, Rosas-Guzman J, Dotta F, Festa A, Zhou M, Kiljanski J (2015). Long-term changes in cardiovascular risk markers during administration of exenatide twice daily or glimepiride: results from the European exenatide study. Cardiovasc Diabetol..

[7] Trahair LG, Horowitz M, Hausken T, Feinle-Bisset C, Rayner CK, Jones KL (2014). Effects of exogenous glucagon-like peptide-1 on the blood pressure, heart rate, mesenteric blood flow, and glycemic responses to intraduodenal glucose in healthy older subjects. J Clin Endocrinol Metab.

[8] Vilsboll T, Christensen M, Junker AE, Knop FK, Gluud LL (2012). Effects of glucagon-like peptide-1 receptor agonists on weight loss: systematic review and meta-analyses of randomised controlled trials. BMJ.

[9] Ussher JR, Drucker DJ (2014). Cardiovascular actions of incretin-based therapies. Circ Res.

[10] Skov J, Pedersen M, Holst JJ, Madsen B, Goetze JP, Rittig S, Jonassen T, Frokiaer J, Dejgaard A, Christiansen JS (2016). Short-term effects of liraglutide on kidney function and vasoactive hormones in type 2 diabetes: a randomized clinical trial. Diabetes Obes Metab.

[11] Asmar A, Simonsen L, Asmar M, Madsbad S, Holst JJ, Frandsen E, Moro C, Sorensen CM, Jonassen T, Bulow J (2016). Glucagon-like peptide-1 does not have acute effects on central or renal hemodynamics in patients with type 2 diabetes without nephropathy. Am J Physiol Endocrinol Metab..

[12] Zhou X, Huang CH, Lao J, Pocai A, Forrest G, Price O, Roy S, Kelley DE, Sullivan KA, Forrest MJ (2015). Acute hemodynamic and renal effects of glucagon-like peptide 1 analog and dipeptidyl peptidase-4 inhibitor in rats. Cardiovasc Diabetol..

[13] Cooney MT, Vartiainen E, Laatikainen T, Juolevi A, Dudina A, Graham IM (2010). Elevated resting heart rate is an independent risk factor for cardiovascular disease in healthy men and women. Am Heart J.

[14] Perret-Guillaume C, Joly L, Benetos A (2009). Heart rate as a risk factor for cardiovascular disease. Prog Cardiovasc Dis.

[15] Robinson LE, Holt TA, Rees K, Randeva HS, O’Hare JP (2013). Effects of exenatide and liraglutide on heart rate, blood pressure and body weight: systematic review and meta-analysis. BMJ Open..

[16] Meier JJ (2012). GLP-1 receptor agonists for individualized treatment of type 2 diabetes mellitus. Nat Rev Endocrinol..

[17] Meier JJ, Rosenstock J, Hincelin-Mery A, Roy-Duval C, Delfolie A, Coester HV, Menge BA, Forst T, Kapitza C (2015). Contrasting effects of lixisenatide and liraglutide on postprandial glycemic control, gastric emptying, and safety parameters in patients with type 2 diabetes on optimized insulin glargine with or without metformin: a randomized, open-label trial. Diabetes Care.

[18] Nakatani Y, Kawabe A, Matsumura M, Aso Y, Yasu T, Banba N, Nakamoto T (2016). Effects of GLP-1 receptor agonists on heart rate and the autonomic nervous system using Holter electrocardiography and power spectrum analysis of heart rate variability. Diabetes Care.

[19] Linnebjerg H, Seger M, Kothare PA, Hunt T, Wolka AM, Mitchell MI (2011). A thorough QT study to evaluate the effects of single dose exenatide 10 microgram on cardiac repolarization in healthy subjects. Int J Clin Pharmacol Ther.

[20] Eli Lilly Canada Inc. Byetta Product Monograph. 2011. http://www.glucagon.com/pdfs/ByettaCanadaPM_11Jan2011_pswd.pdf. Accessed 5 Aug 2016.

[21] Gill A, Hoogwerf BJ, Burger J, Bruce S, Macconell L, Yan P, Braun D, Giaconia J, Malone J (2010). Effect of exenatide on heart rate and blood pressure in subjects with type 2 diabetes mellitus: a double-blind, placebo-controlled, randomized pilot study. Cardiovasc Diabetol..

[22] Tillner J, Golor G, Voirot P, Lehmann A, Frosio C, Megard C, Lorenz M (2016). Lixisenatide (Lyxumia^®^) has no effect on QTc interval in healthy subjects: a thorough QTc study. Diabetes.

[23] Novo Nordisk Canada Inc. Victoza Product Monograph. 2011. http://www.novonordisk.ca/content/dam/Canada/AFFILIATE/www-novonordisk-ca/OurProducts/PDF/victoza-product-monograph.pdf. Accessed 5 Aug 2016.

[24] Endocrinologic and Metabolic Drugs Advisory Committee Meeting: FDA briefing document, NDA206321, Liraglutide injection 3 mg, Sponsor Novo Nordisk. 2014. http://www.fda.gov/downloads/AdvisoryCommittees/CommitteesMeetingMaterials/Drugs/EndocrinologicandMetabolicDrugsAdvisoryCommittee/UCM413317.pdf. Accessed 5 Aug 2016.

[25] van Can J, Sloth B, Jensen CB, Flint A, Blaak EE, Saris WH (2014). Effects of the once-daily GLP-1 analog liraglutide on gastric emptying, glycemic parameters, appetite and energy metabolism in obese, non-diabetic adults. Int J Obes (Lond)..

[26] Lovshin JA, Barnie A, DeAlmeida A, Logan A, Zinman B, Drucker DJ (2015). Liraglutide promotes natriuresis but does not increase circulating levels of atrial natriuretic peptide in hypertensive subjects with type 2 diabetes. Diabetes Care.

[27] Darpo B, Sager P, Macconell L, Cirincione B, Mitchell M, Han J, Huang W, Malloy J, Schulteis C, Shen L, Porter L (2013). Exenatide at therapeutic and supratherapeutic concentrations does not prolong the QTc interval in healthy subjects. Br J Clin Pharmacol.

[28] Sager P, Darpo B, Han J, Kothare P, Linnebjerg H, Mitchell M, Porter L (2011). Exenatide once weekly did not affect corrected QT interval in patients with type 2 diabetes. Diabetes.

[29] Darpo B, Zhou M, Matthews J, Zhi H, Young MA, Perry C, Reinhardt RR (2014). Albiglutide does not prolong QTc interval in healthy subjects: a thorough ECG study. Diabetes Ther..

[30] Ferdinand KC, White WB, Calhoun DA, Lonn EM, Sager PT, Brunelle R, Jiang HH, Threlkeld RJ, Robertson KE, Geiger MJ (2014). Effects of the once-weekly glucagon-like peptide-1 receptor agonist dulaglutide on ambulatory blood pressure and heart rate in patients with type 2 diabetes mellitus. Hypertension.

[31] Ceriello A, Novials A, Canivell S, La SL, Pujadas G, Esposito K, Testa R, Bucciarelli L, Rondinelli M, Genovese S (2014). Simultaneous GLP-1 and insulin administration acutely enhances their vasodilatory, antiinflammatory, and antioxidant action in type 2 diabetes. Diabetes Care.

[32] Kim M, Platt MJ, Shibasaki T, Quaggin SE, Backx PH, Seino S, Simpson JA, Drucker DJ (2013). GLP-1 receptor activation and Epac2 link atrial natriuretic peptide secretion to control of blood pressure. Nat Med.

[33] Pyke C, Heller RS, Kirk RK, Orskov C, Reedtz-Runge S, Kaastrup P, Hvelplund A, Bardram L, Calatayud D, Knudsen LB (2014). GLP-1 receptor localization in monkey and human tissue: novel distribution revealed with extensively validated monoclonal antibody. Endocrinology.

[34] Asmar A, Simonsen L, Asmar M, Madsbad S, Holst JJ, Frandsen E, Moro C, Jonassen T, Bulow J (2015). Renal extraction and acute effects of glucagon-like peptide-1 on central and renal hemodynamics in healthy men. Am J Physiol Endocrinol Metab..

[35] Mendis B, Simpson E, MacDonald I, Mansell P (2012). Investigation of the haemodynamic effects of exenatide in healthy male subjects. Br J Clin Pharmacol.

[36] Griffioen KJ, Wan R, Okun E, Wang X, Lovett-Barr MR, Li Y, Mughal MR, Mendelowitz D, Mattson MP (2011). GLP-1 receptor stimulation depresses heart rate variability and inhibits neurotransmission to cardiac vagal neurons. Cardiovasc Res.

[37] Plamboeck A, Veedfald S, Deacon CF, Hartmann B, Vilsboll T, Knop FK, Holst JJ (2015). The role of efferent cholinergic transmission for the insulinotropic and glucagonostatic effects of GLP-1. Am J Physiol Regul Integr Comp Physiol.

[38] Hillis GS, Hata J, Woodward M, Perkovic V, Arima H, Chow CK, Zoungas S, Patel A, Poulter NR, Mancia G, Williams B, Chalmers J (2012). Resting heart rate and the risk of microvascular complications in patients with type 2 diabetes mellitus. J Am Heart Assoc..

[39] Hillis GS, Woodward M, Rodgers A, Chow CK, Li Q, Zoungas S, Patel A, Webster R, Batty GD, Ninomiya T, Mancia G, Poulter NR, Chalmers J (2012). Resting heart rate and the risk of death and cardiovascular complications in patients with type 2 diabetes mellitus. Diabetologia.

[40] Swedberg K, Komajda M, Bohm M, Borer JS, Ford I, Dubost-Brama A, Lerebours G, Tavazzi L (2010). Ivabradine and outcomes in chronic heart failure (SHIFT): a randomised placebo-controlled study. Lancet.

[41] Voors AA, van der Horst I (2011). Diabetes: a driver for heart failure. Heart.

[42] Bertoni AG, Hundley WG, Massing MW, Bonds DE, Burke GL, Goff DC (2004). Heart failure prevalence, incidence, and mortality in the elderly with diabetes. Diabetes Care.

[43] Ryden L, Thrainsdottir I, Swedberg K (2007). Adjudication of serious heart failure in patients from PROactive. Lancet.

[44] Komajda M, McMurray JJ, Beck-Nielsen H, Gomis R, Hanefeld M, Pocock SJ, Curtis PS, Jones NP, Home PD (2010). Heart failure events with rosiglitazone in type 2 diabetes: data from the RECORD clinical trial. Eur Heart J.

[45] Scirica BM, Bhatt DL, Braunwald E, Steg PG, Davidson J, Hirshberg B, Ohman P, Frederich R, Wiviott SD, Hoffman EB, Cavender MA, Udell JA, Desai NR, Mosenzon O, McGuire DK, Ray KK, Leiter LA, Raz I (2013). Saxagliptin and cardiovascular outcomes in patients with type 2 diabetes mellitus. N Engl J Med.

[46] Kumarathurai P, Anholm C, Nielsen OW, Kristiansen OP, Molvig J, Madsbad S, Haugaard SB, Sajadieh A (2016). Effects of the glucagon-like peptide-1 receptor agonist liraglutide on systolic function in patients with coronary artery disease and type 2 diabetes: a randomized double-blind placebo-controlled crossover study. Cardiovasc Diabetol..

[47] Margulies KB, Anstrom KJ, Hernandez AF, Redfield MM, Shah MR, Braunwald E, Cappola TP (2014). GLP-1 agonist therapy for advanced heart failure with reduced ejection fraction: design and rationale for the functional impact of GLP-1 for heart failure treatment study. Circ Heart Fail..

[48] Margulies KB, Hernandez AF, Redfield MM, Givertz MM, Oliveira GH, Cole R, Mann DL, Whellan DJ, Kiernan MS, Felker GM, McNulty SE, Anstrom KJ, Shah MR, Braunwald E, Cappola TP (2016). Effects of liraglutide on clinical stability among patients with advanced heart failure and reduced ejection fraction: a randomized clinical trial. JAMA.

[49] Jorsal A, Wiggers H, Holmager P, Nilsson B, Nielsen R, Boesgaard TW, Kumme A, Moller JE, Videbaek L, Kistorp C, Gustafsson I, Tarnow L, Flyvbjerg A (2014). A protocol for a randomised, double-blind, placebo-controlled study of the effect of LIraglutide on left VEntricular function in chronic heart failure patients with and without type 2 diabetes (The LIVE Study). BMJ Open..

[50] Jorsal A, Kistorp C, Holmager P, Tougaard RS, Nielsen R, Hanselmann A, Nilsson B, Moller JE, Hjort J, Rasmussen J, Boesgaard TW, Schou M, Videbaek L, Gustafsson I, Flyvbjerg A, Wiggers H, Tarnow L (2017). Effect of liraglutide, a glucagon-like peptide-1 analogue, on left ventricular function in stable chronic heart failure patients with and without diabetes (LIVE)-a multicentre, double-blind, randomised, placebo-controlled trial. Eur J Heart Fail.

[51] Marso SP, Daniels GH, Brown-Frandsen K, Kristensen P, Mann JF, Nauck MA, Nissen SE, Pocock S, Poulter NR, Ravn LS, Steinberg WM, Stockner M, Zinman B, Bergenstal RM, Buse JB (2016). Liraglutide and cardiovascular outcomes in type 2 diabetes. N Engl J Med.

[52] Pfeffer MA, Claggett B, Diaz R, Dickstein K, Gerstein HC, Kober LV, Lawson FC, Ping L, Wei X, Lewis EF, Maggioni AP, McMurray JJ, Probstfield JL, Riddle MC, Solomon SD, Tardif JC (2015). Lixisenatide in patients with type 2 diabetes and acute coronary syndrome. N Engl J Med.

[53] Holman RR, Paul SK, Bethel MA, Matthews DR, Neil HA (2008). 10-year follow-up of intensive glucose control in type 2 diabetes. N Engl J Med.

